# Impact of Helicobacter pylori Eradication on Surgical Treatment of Peptic Ulcer Disease: Systematic Review

**DOI:** 10.7759/cureus.63523

**Published:** 2024-06-30

**Authors:** Suhad A Aljuhani, Ahmad A Sherwani, Fahad O Alnamshah, Rana A Alaeq, Husain A Alrahma, Mada M Jarad, Arwa A Hakami, Tasneem H Mobarki, Hamood K Al-Khairat, Yasir A Sahal, Abdulelah W Bakhsh

**Affiliations:** 1 General Surgery, Al Thager Hospital, Jeddah, SAU; 2 General Surgery, King Fahad Central Hospital, Jazan, SAU; 3 Internal Medicine, Rijal Alma Hospital, Abha, SAU; 4 Medical Laboratories Technology, Faculty of Applied Medical Sciences, Taibah University, Medina, SAU; 5 Gastroentrology and Hepatology, Salmaniya Medical Complex, Manama, BHR; 6 General Surgery, Health Affairs in Jazan Region, Jazan, SAU; 7 General Surgery, Ministry of Health, Jazan, SAU

**Keywords:** stomach, surgery, eradication, helicobacter pylori, peptic ulcer disease

## Abstract

Peptic ulcer disease (PUD) poses a significant global healthcare challenge, with an intricate interplay between stomach acid-pepsin levels and mucosal protective mechanisms. The emergence of Helicobacter pylori (H. pylori) as a major etiological factor revolutionized the therapeutic landscape, highlighting the importance of bacterial eradication in PUD management. Surgical intervention remains vital, particularly in cases of perforated peptic ulcers, despite a shift towards conservative approaches. Understanding the impact of H. pylori eradication on surgical outcomes is crucial for optimizing PUD management. This systematic review was conducted to assess how H. pylori eradication treatment impacts surgical results in patients with PUD. The criteria for inclusion involved research studies on individuals aged 18 years and older with a diagnosis of PUD that necessitated surgical treatment. Important results comprised eradication rates, complications after surgery, recurrence rates, and overall outcomes for patients. Two researchers independently screened and extracted data from electronic databases using a thorough search strategy. The evaluation of quality employed standardized instruments for randomized controlled trials and cohort studies. Nine research projects met the requirements for inclusion, offering information on the effectiveness of H. pylori elimination treatment on surgical results. Different rates of eradication were noted, with a notable number of patients experiencing postoperative complications. Recurrence of ulcers was a concern, despite treatment, emphasizing the complexity of PUD management. Studies have shown that eradication therapy is effective in certain patient groups, like young men with perforated peptic ulcers. Still, there are obstacles, especially for patients who test negative for H. pylori and experience recurring ulcers. The integration of H. pylori eradication with surgical intervention represents a holistic approach to PUD management. Although eradication therapy has the potential to enhance surgical results, difficulties remain, requiring personalized treatment approaches that consider patients' unique characteristics and the cause of the disease. This research adds to the growing knowledge of PUD treatment, stressing the importance of proper management.

## Introduction and background

Peptic ulcer disease (PUD) is a major healthcare problem globally, arising from a complex interaction between stomach acid-pepsin levels and the protective functions of the mucosal lining. Mainly impacting the stomach and upper part of the small intestine, PUD puts a significant strain on healthcare systems worldwide. PUD is characterized by a wide range of clinical symptoms and complications, with a lifetime prevalence estimated to be between 5% and 10% and an annual incidence of 0.1% to 0.3% in Western nations [1,2]. Peptic ulcers in the duodenum or stomach typically start without symptoms and can develop into various clinical manifestations such as mild dyspepsia, gastrointestinal bleeding, perforation, and gastric outlet obstruction [3].

Perforation in peptic ulcers is a serious complication, impacting around 2% to 10% of PUD patients and with a mortality rate of nearly 20% [4,5]. Early surgical intervention coupled with aggressive sepsis management remains the cornerstone of therapy for perforated peptic ulcers [6]. Nevertheless, the treatment options for PUD drastically changed when Helicobacter pylori (H. pylori), a gram-negative bacterium, was identified as a key factor in its development [7]. Before this discovery, excessive acid secretion was commonly thought to be the main cause of PUD. Nonetheless, the presence of H. pylori in the stomach lining revealed a complicated connection between bacterial infection, inflammation of the mucosa, and the development of ulcers.

Colonization by H. pylori causes inflammation in the stomach lining, disturbing the fragile balance of defense mechanisms in the mucosa. This bacterium is a major cause of gastric and duodenal ulcers worldwide. Furthermore, H. pylori infection has effects that go beyond the digestive system, with links observed in acute and chronic gastritis, gastric adenocarcinoma, gastric lymphoma, and other non-digestive diseases [8]. Consequently, H. pylori is seen as a crucial approach in treating PUD, helping with ulcer healing and reducing the risk of recurrence. Many research studies have highlighted the effectiveness of eliminating H. pylori in treating simple peptic ulcers and decreasing the chance of repeated bleeding after endoscopic treatment [9]. Both the National Institute of Health Consensus Meeting and the Maastricht Meeting of the European Helicobacter pylori Study Group agree on recommending H. pylori eradication as the standard treatment for uncomplicated and bleeding peptic ulcers. This highlights the crucial importance of eliminating H. pylori in changing treatment strategies and improving results in managing PUD [10,11].

Modern guidelines recommend a more cautious surgical approach in treating PUD, focusing on medical and endoscopic treatments before considering surgery. Surgery is mainly used for cases that do not respond to medical or endoscopic treatments or for rare complications like free perforation, refractory bleeding, or gastric outlet obstruction. In addition, surgery may be necessary if multiple attempts to eliminate the H. pylori bacteria have not been successful or if there are recurring ulcers that are not caused by H. pylori [12-15]. Moreover, the decrease in PUD-related surgeries in recent years highlights the effectiveness of non-surgical treatments in treating this condition.

Incorporating H. pylori eradication into surgical interventions is a comprehensive strategy to decrease the necessity for invasive procedures and improve patient results. This study aims to offer valuable insights into the current management landscape of PUD by explaining how surgery's role has changed in light of medical advancements. It is important to comprehend the impact of eradicating H. pylori on the need, timing, and results of surgical procedures to improve patient care and healthcare resource management. Additionally, examining how H. pylori eradication can be combined with surgical methods provides insight into the combined advantages and difficulties in treating complicated cases of PUD. Therefore, this study aims to add to the current discussion on PUD treatment and aid in clinical decision-making for better patient results.

## Review

Materials and methods

Definition of Outcomes and Inclusion Criteria

The results evaluated in this study focus on the effect of H. pylori eradication treatment affects surgical outcomes for patients with peptic ulcer disease. These results include various aspects related to the surgical treatment of peptic ulcers and the effects of H. pylori eradication on patient outcomes including eradication rate, postoperative complications, recurrence rates, and mortality outcomes. Published studies meeting the criteria for this review include those that involved human participants, specifically individuals aged 18 years or older who have been diagnosed with peptic ulcer disease and require surgery. Thesestudies focus on analyzing the impact of H. pylori eradication treatment before surgical procedures. Appropriate research methods include randomized controlled trials (RCTs), prospective cohort studies, and retrospective cohort studies. Moreover, qualifying studies should include data on pertinent surgical and clinical results, including success rates of surgery, postoperative issues, peptic ulcer recurrence, and long-term patient monitoring. Additionally, only research conducted in the English language is taken into account for inclusion. To ensure the accuracy and importance of the results, research that did not meet the specified criteria was excluded. Additionally, case studies, conference summaries, editorials, opinion pieces, and research performed before 2000 were not included. Individuals who had peptic ulcer surgery in the past, pregnant patients, or those with notable conditions found during endoscopy other than gastric or duodenal ulcers were also not included.

Search Strategy

We carried out an extensive search on various electronic databases such as PubMed, Scopus, Web of Science, and ScienceDirect to find research studies. The review used specific keywords such as (Helicobacter pylori, Campylobacter pylori, Campylobacter pyloridis, H. pylori) and (surgical treatment, operative procedure, operative surgical procedures, operative surgical procedures) and (peptic ulcer disease, stomach ulcer, gastric ulcer, duodenal ulcer, gastroduodenal ulcer, gastroduodenal ulcers, gastroduodenal ulcers) in the search strategy. This all-encompassing strategy aimed to discover relevant research examining how eliminating H. pylori affects surgical results in patients with peptic ulcer disease.

Screening and extraction

Articles with irrelevant titles were excluded from consideration. In the subsequent phase, both the full text and abstracts of the papers were meticulously reviewed to determine their compliance with the inclusion criteria. To streamline the process, titles, and abstracts were organized, assessed, and scrutinized for any duplicate entries using reference management software Endnote X8 (2013, Clarivate, Philadelphia, PA). To ensure the highest quality of selection, a dual screening approach was adopted, involving one screening for the evaluation of titles and abstracts, and another for the comprehensive examination of the entire texts. Once all relevant articles were identified, a structured extraction sheet was created to capture pertinent information aligned with our specific objectives.

Two separate researchers conducted the data extraction process independently. The gathered information included various study attributes like the author's name, publication year, country of origin, study design, gender, and sample size.

Quality Assessment

We utilized the Cochrane risk of bias assessment tool to assess the methodological quality and bias risk in RCTs examined in this systematic review [16]. In addition, we used the Newcastle-Ottawa Scale (NOS) to evaluate the quality and possible biases in cohort studies [17].

Results

Search Results

**Figure 1:**
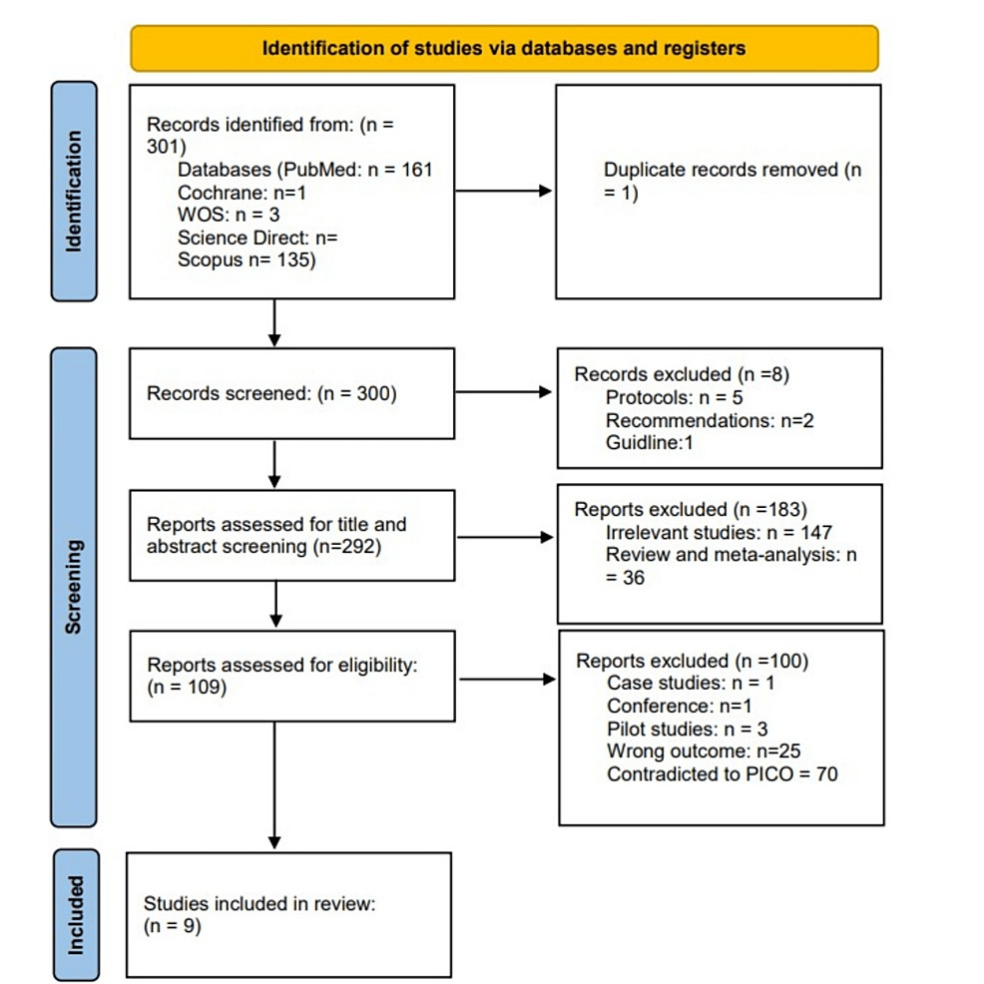
PRISMA flow diagram WOS: Web of Science; PICO: patient/population, intervention, comparison, and outcomes

We executed the search methodologies outlined previously, resulting in the identification of a total of 301 citations, subsequently, one duplicated study was deleted. Upon screening titles and abstracts, only 109 citations met the eligibility criteria for further consideration. The screening resulted in nine studies [18-26] meeting the inclusion criteria outlined in the methodology section. These studies were chosen for their importance in assessing how the treatment of Helicobacter pylori affects surgical results in peptic ulcer patients. Figure 1 provides an in-depth depiction of the search strategy and screening process.

Results of Quality Assessment

Quality assessment of cohort studies using NOS: The evaluation considered multiple criteria including the representativeness of the exposed cohort, selection of the non-exposed cohort, ascertainment of exposure, comparability of cohorts, and assessment of outcome. Each study was classified into high, moderate, or low-quality levels depending on the overall evaluation of these criteria. Table 1 shows that four studies were of high quality, while three studies were of moderate quality.

**Table 1:** Quality assessment of cohort studies using Newcastle-Ottawa Scale A study can be awarded a maximum of one star for each item within the selection and outcome categories. A maximum of two stars can be given for comparability.

Study name	Representativeness of the exposed cohort (★)	Selection of the non-exposed cohort (★)	Ascertainment of exposure (★)	Demonstration that outcome of interest was not present at the start of the study (★)	Comparability of cohorts based on of the design or analysis (max★★)	Assessment of outcome (★)	Was follow-up long enough for outcomes to occur? (★)	Adequacy of follow-up of cohorts (★)	Quality level
Dodiyi-Manuel et al. [18]	★	★	★	★	★	★	★	★	High
Hasadia et al. [19]	★	-	★	★	★	★	★	-	Moderate
Rasane et al. [20]	★	-	★	★	★	★	★	★	High
Smith et al. [21]	★	★	★	★	★	★	★	-	Moderate
Chalya et al. [23]	★	-	★	★	★★	★	★	-	High
El-Nakeeb et al. [24]	★	★	★	-	★★	★	★	-	High
Dalcin et al. [26]	★	-	★	★	★	★	★	-	Moderate

**Figure 2:**
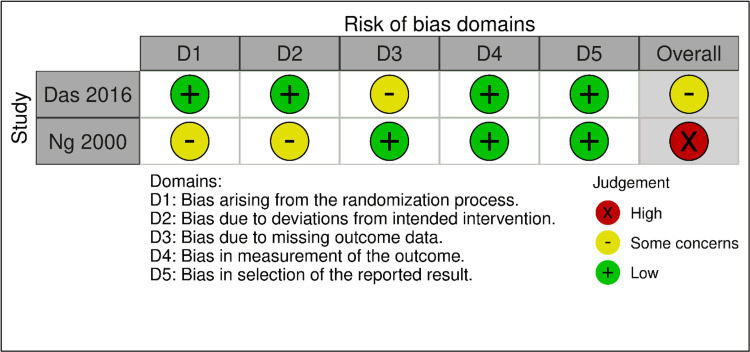
Risk of bias assessment of randomized controlled trials using Rob-2 tool.

Risk of bias assessment of randomized controlled trials using Rob-2 tool: The assessment focused on key domains including bias arising from the randomization process, deviations from intended interventions, missing outcome data, measurement of the outcome, and selection of the reported result. The figure illustrates the level of bias identified across different RCTs included in the review (Figure 2).

Characteristics of the Included Studies

This review included nine studies with 662 participants, conducted between 2009 and 2019, covering different geographical areas and research methodologies. It is worth mentioning that most of the research was conducted using cohort designs (six studies), while three RCTs were also part of the study. In terms of study location, the research was spread out in different parts, including two studies from Egypt, Tanzania, and the USA, along with one study each from Nigeria, India, China, and Brazil (Table 2).

**Table 2:** Baseline characteristics of included studies NR: Not reported; RCT: randomized controlled trials

Study ID	Country	Design	Sample size	Age, mean (SD)	Female/Male%	History of H. pylori, n (%)	History of previous ulcer, n(%)
Dodiyi-Manuel et al. 2015 [18]	Nigeria	Cohort	36	42.1 (12.3)	22.3/77.7	NR	24 (55.6)
Hasadia et al. 2018 [19]	Israel	Cohort	81	Age group: ≥65 years 32.50%	13.6/86.4	25 (38.5)	21 (25.9)
Rasane et al. 2019 [20]	USA	Cohort	79	56.89 (16.51)	53.16/46.84	42 (53.2)	NR
Smith et al. 2017 [21]	USA	Cohort	94	NR	47.9/52.1	10 (11)	NR
Das et al. 2016 [22]	India	RCT	105	sequential: 44.11 (13.75), concomitant: 42.45 (13.65)	sequential: 91/9%, concomitant: 88/12%	68 (64.76)	NR
Chalya et al. 2011 [23]	Tanzania	Cohort	84	26 (6.8)	42.9/57.1	NR	26 (31)
El-Nakeeb et al. 2009 [24]	Egypt	Cohort	83	47.8 (7.2)	18/82	65 (84.4)	NR
Ng et al. 2000 [25]	China	RCT	99	52.2 (18.1)	15/85	80 (81)	25 (25)
Dalcin et al. 2009 [26]	Brasil	Cohort	51	47.3 (14.93)	20/80	25 (49)	NR

Study Outcome Measures

The results of the included studies shed light on the efficacy of H. pylori eradication therapy on surgical outcomes in patients with peptic ulcer disease. Across the studies, the eradication rates varied, with some studies reporting high rates following treatment. Postoperative complications were observed in a significant proportion of patients, with mortality rates ranging from moderate to high. Notably, the recurrence of ulcers was a concern, with a substantial number of patients experiencing recurrent ulcers despite treatment (Table 3).

**Table 3:** Outcome measures of the included studies NR: Not Reported; CT: Concomitant Therapy; ST: Sequential Therapy

Study ID	Eradication rate	Complication rate	Ulcer recurrence	Mortality	Surgery outcomes
	n(%)	n(%)	n(%)	n(%)	
Dodiyi-Manuel et al. 2015 [18]	NR	13(36.11%)	NR	4 (11.11%)	Young men were the majority of those affected with perforated peptic ulcers, and the most successful treatment method was the removal of H. pylori followed by Graham's omental patch.
Hasadia et al. 2018 [19]	NR	3(3.75%)	3 (3.75%)	16 (20.00%)	After surgery for peptic ulcer disease complications, mortality is significant; this is especially true for patients having surgery to treat bleeding. Even after undergoing procedures to eliminate stomach acid secretion and eradicate the H. pylori bacteria, patients frequently require repeat surgeries and hospital stays due to problems.
Rasane et al. 2019 [20]	NR	NR	8 (10.13%)	3 (3.80%)	Clinically, H. pylori-negative patients fared worse than H. pylori-positive individuals. These results might indicate a variation in the peptic ulcer disease process's underlying pathophysiology. It is necessary to conduct additional research.
Smith et al. 2017 [21]	NR	7(7.45%)	9 (9.75%)	17(18.09%)	The underlying disease process that causes foregut perforation is not addressed by omental patching, which has a 12% endoscopically verified recurrent ulceration rate and a 23% frequency of recurrent symptoms within 44 months. After 22 months, patients typically quit taking proton pump inhibitors, at which point their risk increases.
Das et al. 2016 [22]	25 in sequential 25(86.21%), 29 in concomitant 29 (96.67%)	NR	5, 2		The two groups' rates of H. pylori eradication, side effects, compliance, cost, and ulcer recurrences were comparable. In terms of economy, the ST outperformed the CT.
Chalya et al. 2011 [23]	NR	25 (29.76%)	NR	9 (10.71%)	In our setting, peptic ulcer perforation is still a common clinical issue that primarily affects young men who are not known to have peptic ulcer disease. Despite the patients' delayed arrival at our center, simple closure with an omental patch followed by H. pylori eradication was effective with great results in the majority of survivors.
El-Nakeeb et al. 2009 [24]	triple therapy: 84.8%, omeprazole: 51.9%	triple therapy:17 (50%), omeprazole:13 (41.94%)	triple therapy:2 (5.88%), omeprazole:8 (25.81%)	NR	A significant percentage of individuals with duodenal ulcer perforation have H. pylori. After a perforated duodenal ulcer was simply closed, the incidence of recurring ulcers was decreased by eliminating H. pylori.
Ng et al. 2000 [25]	NR	NR	18 (23.08%	NR	For patients with perforated duodenal ulcers linked to H. pylori, eradication of the bacteria reduces ulcer recurrence. It is not necessary to do immediate acid-reduction surgery when widespread peritonitis is present.
Dalcin et al. 2009 [26]	NR	NR	12 (24.49%	NR	For the positive group, a straightforward suture combined with H. pylori eradication is the gold standard; for the negative group, the issue of acid-reducing techniques remains open.

Eradication and recurrence rates

The findings from Dodiyi-Manuel et al. [18] underscored that young men predominantly suffered from perforated peptic ulcers, with successful treatment primarily involving H. pylori removal followed by Graham's omental patch. Smith et al. [21] emphasized the persistence of recurrent ulcers post-omental patching, especially after discontinuing proton pump inhibitors. Das et al. [22] found comparable H. pylori eradication rates and ulcer recurrences between sequential and concomitant therapy, with sequential therapy being more economical. Chalya et al. [23] reported effective outcomes with omental patch closure and H. pylori eradication for peptic ulcer perforation in young men. El-Nakeeb et al. [24] highlighted decreased ulcer recurrence post-H. pylori eradication following simple closure of perforated duodenal ulcers. Ng et al. [25] emphasized the role of H. pylori eradication in reducing ulcer recurrence in patients with perforated duodenal ulcers. Dalcin et al. [26] suggested that a straightforward suture combined with H. pylori eradication is effective for H. pylori-positive individuals, while the approach for H. pylori-negative individuals remains debatable.

Complications

Among the included studies the highest incidence of complications 36.11% was reported by Dodiyi-Manuel et al. [18] while the lowest 3.75% was stated by Hasadia et al. [19]. Furthermore, Hasadia et al. [19] highlighted significant mortality following surgery for peptic ulcer disease complications, particularly in cases of bleeding, despite acid secretion reduction and H. pylori eradication. Rasane et al. [20] noted worse outcomes in H. pylori-negative patients, suggesting varied underlying pathophysiology requiring further investigation. These findings collectively underscore the varied efficacy and complexities surrounding H. pylori eradication therapy and surgical outcomes in peptic ulcer disease management, indicating the need for tailored treatment approaches based on individual patient characteristics and disease etiology.

Discussion

This study sought to provide valuable insights into the evolving landscape of PUD management, particularly concerning the changing role of H. pylori eradication. The findings illuminated the impact of H. pylori eradication therapy on surgical outcomes in PUD patients. The efficacy of this therapy varied across included studies, with some reporting high eradication rates post-treatment. The findings from various included studies highlight several key points regarding the management of PUD. Firstly, they indicate that young men are more prone to suffering from perforated peptic ulcers, and successful treatment typically involves removing H. pylori followed by using Graham's omental patch. Additionally, omental patch closure combined with H. pylori eradication proves to be effective for treating peptic ulcer perforation in young men, while simple closure of perforated duodenal ulcers leads to decreased ulcer recurrence after H. pylori eradication. However, it was noted that recurrent ulcers are still common, especially when proton pump inhibitors are discontinued. Moreover, different treatment approaches, such as sequential and concomitant therapy, show similar rates of H. pylori eradication and ulcer recurrence, with sequential therapy being more cost-effective. These studies also emphasize the importance of H. pylori eradication in reducing ulcer recurrence, particularly in patients with perforated duodenal ulcers. However, the efficacy of a straightforward suture combined with H. pylori eradication seems to vary depending on the presence of H. pylori, with its effectiveness being uncertain in H. pylori-negative individuals. Moreover, significant mortality rates are observed following surgery for PUD complications, particularly in cases of bleeding, despite efforts to reduce acid secretion and eradicate H. pylori. Furthermore, worse outcomes are noted in H. pylori-negative patients, suggesting diverse underlying pathophysiology that warrants further investigation. These collective findings underscore the diverse effectiveness and complexities surrounding H. pylori eradication therapy and surgical outcomes in managing PUD, emphasizing the necessity for tailored treatment approaches based on individual patient characteristics and disease etiology.

Similarly, the study conducted by Powel et al. shed light on the substantial cost savings associated with the successful eradication of H. pylori in ulcerative diseases. By effectively eliminating the bacterial infection, the need for prolonged treatment and management of peptic ulcers is reduced, leading to decreased healthcare expenditures [27]. Additionally, findings from studies by Connor et al. and Graham et al. further reinforced the benefits of H. pylori eradication in promoting faster healing of peptic ulcers. By eradicating the underlying cause of the ulcers, H. pylori eradication not only accelerates the healing process but also reduces the risk of ulcer recurrence and associated complications. These findings underscore the economic and clinical significance of H. pylori eradication in the management of peptic ulcer disease, highlighting its potential to improve patient outcomes while simultaneously reducing healthcare costs [28,29].

Conversely, this systematic review also underscored that a significant portion of patients encountered postoperative complications, with mortality rates fluctuating from moderate to high. The rate of postoperative complications in cases where H. pylori tests were positive, was found to be higher than in H. pylori-negative individuals [30]. Moreover, the severity of the post-operative complications was also observed to be worse in those study participants in which H. pylori was positive [31].

For individuals identified as negative for H. pylori strains, the eradication regimen didn't yield improvement, as the underlying cause of the ulcer was non-bacterial in origin. This implies that the efficacy of H. pylori eradication therapy was limited in cases where the etiology of the ulcer was not linked to bacterial infection [32,33]. Consequently, these patients didn't benefit from the treatment approach targeting H. pylori, suggesting the necessity for alternative therapeutic strategies tailored to the specific underlying cause of their ulcers. This aspect highlights the importance of accurately diagnosing the underlying pathology of peptic ulcers to guide appropriate treatment decisions and optimize patient outcomes. Additionally, the presence of postoperative complications and varying mortality rates underscores the complexity of managing PUD surgically, necessitating careful consideration of individual patient factors and disease characteristics in treatment planning [34]. Furthermore, the recurrence of ulcers was also highlighted significant concern, despite treatment efforts [35].

Several research studies have consistently highlighted a higher incidence of perforated peptic ulcers in men compared to women. This gender disparity suggests that men may be at a higher risk for developing gastric issues, particularly perforated peptic ulcers. Conversely, the lower incidence rate in women implies a relative advantage in terms of gastric health. Understanding these gender-based differences in ulcer prevalence can inform targeted prevention and management strategies, potentially leading to improved outcomes and reduced healthcare burden for both men and women affected by peptic ulcer disease [36,37]. The present systematic review also highlighted that a higher rate of perforated ulcers was reported in men as compared to women which endorses the findings from other similar studies and research.

In this systematic review, it was found that the effectiveness of eliminating H. pylori was particularly linked to addressing Graham's omental pouch during surgical interventions. This suggests that when surgeons focused on dealing with Graham's pouch during the procedure, it led to a higher success rate in eradicating the bacterium as demonstrated by findings of two of the included studies. Arora et al. [38] specifically pointed out that the removal of Graham's pouch led to improvements in both ulcer healing and mortality rates. This emphasizes the importance of considering Graham's omental pouch removal as a preferred strategy for managing peptic ulcers, particularly in the context of H. pylori eradication. By focusing on this technique, clinicians can potentially enhance treatment outcomes and reduce mortality associated with peptic ulcer disease. Additionally, these findings contribute to a deeper understanding of the optimal surgical approaches for addressing peptic ulcers and highlight the need for further research to explore the efficacy of different repair techniques in improving patient outcomes [38]. Another approach of using straightforward sutures was also explored in this systematic review, yet it wasn’t proven to be effective for H. pylori-negative individuals. Simple sutures without the removal of Graham’s pouch were found to be an effective strategy for the resolution of peptic ulcers in a prospective study [39].

The systematic review also underscored the significance of ulcer recurrence and the various factors influencing this phenomenon. A key finding was that discontinuing proton pump inhibitors emerged as a major risk factor for ulcer recurrence, particularly following Graham's pouch removal. Evidence has revealed that ulcers tended to recur, especially after omental patching, with discontinuation of proton pump inhibitors being a contributing factor. This implies that the cessation of proton pump inhibitors post-surgery is associated with an increased risk of ulcer recurrence, particularly in cases where Graham's pouch removal has been performed [40,41]. The evidence also suggests a correlation between the discontinuation of proton pump inhibitors and the recurrence of ulcers following omental patching, highlighting the importance of continued acid suppression therapy in preventing ulcer recurrence post-surgery. This finding underscores the critical role of proton pump inhibitors in the long-term management of peptic ulcers, particularly after surgical intervention. Clinicians should be mindful of this risk factor and consider appropriate strategies to ensure continued acid suppression therapy in patients undergoing omental patching or other surgical procedures for peptic ulcers. By addressing this risk factor and optimizing acid suppression therapy, clinicians can potentially reduce the likelihood of ulcer recurrence and improve long-term outcomes for patients with peptic ulcer disease [41,42].

Recent trends in post-surgery mortality rates for peptic ulcers have raised serious concerns. Despite efforts to address contributing factors such as reducing acid secretion and eradicating H. pylori, evidence indicates that these interventions have not significantly influenced mortality levels, especially in cases involving bleeding. This suggests that factors beyond acid secretion and H. pylori presence or eradication significantly impact the healing process and mortality outcomes in peptic ulcer cases [43]. The observation underscores the critical role of the ulcer's underlying pathophysiology in determining its healing trajectory, independent of the influence of known risk factors. In essence, while interventions targeting acid secretion reduction and H. pylori eradication are crucial components of peptic ulcer treatment, their efficacy in reducing mortality rates may be limited in cases where bleeding occurs [44]. This highlights the complexity of peptic ulcer disease and the need for a nuanced understanding of its underlying mechanisms to improve patient outcomes effectively. By recognizing the multifaceted nature of peptic ulcer pathophysiology, clinicians can better tailor treatment strategies to address specific patient needs and optimize healing outcomes. Additionally, this insight underscores the importance of ongoing research to elucidate the diverse factors contributing to peptic ulcer development and progression, ultimately guiding the development of more effective therapeutic approaches [45].

Moreover, the current systematic review highlighted that the efficacy of H. pylori eradication varied depending on the type of therapy utilized. Various studies indicated that concomitant therapy emerged as a preferred option for H. pylori eradication based on the obtained results [46]. Nevertheless, sequential therapy also demonstrated comparable effectiveness in eliminating the bacteria, alongside the added advantage of being more economical [22]. These findings suggest that both concomitant and sequential therapy regimens offer viable approaches for eradicating H. pylori infection, albeit with differing cost implications. Multiple investigations supported concomitant therapy as an optimal strategy for achieving successful H. pylori eradication. However, it's important to note that sequential therapy also yielded favorable outcomes in terms of bacterial eradication.

Furthermore, sequential therapy was identified as a more cost-effective option compared to concomitant therapy. This implies that while concomitant therapy may be preferred in certain clinical scenarios, sequential therapy presents a viable alternative that can achieve comparable results at a lower cost. The observed variation in H. pylori eradication rates between different therapeutic approaches underscores the importance of tailoring treatment regimens to individual patient needs and resource constraints [22]. Clinicians should consider factors such as treatment efficacy, cost-effectiveness, and patient preferences when selecting the most appropriate therapy for H. pylori eradication. By evaluating the pros and cons of each approach, healthcare providers can optimize treatment outcomes while also ensuring efficient resource allocation in the management of H. pylori infection. The study by Das et al. [22] included in the current systematic review found the eradication rates of H. pylori similar between both the concomitant and sequential therapies, however, economically, sequential therapy was estimated to be more cost-effective.

Strengths and limitations

One of the strengths of the review lies in its comprehensive approach to synthesizing evidence from multiple studies, providing a holistic understanding of the impact of H. pylori eradication on surgical outcomes in peptic ulcer disease. By including a diverse range of studies, the review captures a broad spectrum of findings, enhancing the generalizability of its conclusions. Additionally, the review's focus on surgical treatment adds depth to the existing literature, shedding light on an important aspect of peptic ulcer management that may be overlooked in studies focusing solely on medical interventions. Furthermore, the review's attention to various factors influencing surgical outcomes, such as patient demographics, underlying pathophysiology, and treatment modalities, enriches its analysis and allows for a nuanced interpretation of the findings. This comprehensive approach enables clinicians to better understand the complexities surrounding peptic ulcer disease management and tailor treatment strategies accordingly.

However, the review also has several limitations that warrant consideration. Firstly, the heterogeneity among the included studies in terms of study design, patient population, and outcome measures may introduce bias and complicate the interpretation of results. Additionally, the quality of evidence in some studies may be variable, potentially affecting the robustness of the review's conclusions. Lastly, the review's inability to control for confounding variables and assess causality limits its ability to draw definitive conclusions about the efficacy of H. pylori eradication in improving surgical outcomes for peptic ulcer disease. Despite these limitations, the systematic review provides valuable insights into the complexities of peptic ulcer management and highlights areas for future research and clinical practice improvement.

Future direction

Moving forward, future research in the field of peptic ulcer disease management should aim to address several key areas to further enhance patient outcomes. Firstly, there is a need for well-designed prospective studies that systematically evaluate the efficacy of H. pylori eradication therapy in conjunction with surgical interventions. These studies should employ standardized protocols for both H. pylori eradication and surgical procedures to minimize variability and facilitate comparisons across different treatment modalities. In addition to that, there is a need for comparative effectiveness research to assess the relative merits of different H. pylori eradication regimens, such as concomitant therapy versus sequential therapy, in the context of surgical management. This research should consider not only the efficacy of bacterial eradication but also factors such as cost-effectiveness, tolerability, and patient adherence. Efforts should be made to explore the role of adjunctive therapies, such as proton pump inhibitors and mucosal protectants, in preventing ulcer recurrence and reducing postoperative complications. 

## Conclusions

This review comprehensively highlighted the impact of eradication of the H. Pylori on the surgical treatment of PUD. Our findings note diverse rates of eradication along with significant incidence of post-operative complications. Moreover, the incidence of recurrence of ulcers further highlighted the complexity of the management of PUD. This signifies that although eradication therapy may improve surgical outcomes, the risk of complications persists hence a tailored therapeutic approach is needed. Research in future should prioritize the development of personalized treatment approaches that take into account individual patient characteristics, disease severity, and underlying pathophysiology to optimize outcomes and improve the quality of life for patients with PUD.
